# Effects of the Salt-Processing Method on the Pharmacokinetics and Tissue Distribution of Orally Administered *Morinda officinalis* How. Extract

**DOI:** 10.1155/2020/5754183

**Published:** 2020-02-11

**Authors:** Ji Shi, Xiaohang Ren, Jia Wang, Xiaofeng Wei, Bonan Liu, Tianzhu Jia

**Affiliations:** School of Pharmacy, Liaoning University of Traditional Chinese Medicine, Dalian 116600, China

## Abstract

Salt processing, which involves steaming with salt water, directs herbs into the kidney channel. After being salt processed, kidney invigorating effects occur. However, the underlying mechanism of this method remains elusive. The compounds monotropein, rubiadin, and rubiadin 1-methyl ether are the major effective components of *Morinda officinalis* How. To clarify the pharmacokinetics and tissue distribution of these three compounds, we employed liquid chromatography-tandem mass spectrometry (UPLC-MS/MS) to determine the contents of the three components in rat plasma and tissues. Separation was achieved on an Acquity UPLC HSS T3 column (100 mm × 2.1 mm, 1.8 *μ*m, Waters). Formic acid aqueous solution (0.1%; A) and acetonitrile (containing 0.1% formic acid; B) were used as the mobile phase system with a programmed elution of 0∼5 min with 70% A and then 5∼7 min with 60% A. All analytes were measured with optimized multiple reaction monitoring (MRM) in negative ion mode. Geniposide and 1,8-dihydroxyanthraquinone were used as the internal standards (IS). The linear ranges were 1.2∼190, 1.3∼510, and 0.047∼37.5 *μ*g/mL, respectively. Compared with the *Morinda officinalis* without wood (MO) group, the C_ma_x and AUC_0-t_ parameters of rubiadin and rubiadin 1-methyl ether elevated remarkably for the salt-processed *Morinda officinalis* (SMO) groups, which indicates that steaming by salt could increase the bioavailability of rubiadin and rubiadin 1-methyl ether. The *T*_max_ for monotropein is shorter (0.5 h) in SMO groups than that in MO group, which means that monotropein was quickly absorbed in the SMO extract. Moreover, the contents of three compounds in the small intestine were the highest.

## 1. Introduction

*Morinda officinalis* is a commonly used traditional Chinese medicines (TCMs) [1], which has been used in China for many years. People use it as a tonic or tonifying kidney product to protect against and cure depression, rheumatoid arthritis, osteoporosis, and kidney-yang deficiency syndrome [2–6]. Its main active components include oligosaccharides, polysaccharides, iridoid glycosides, and anthraquinones [7–9].

Chinese medicinal herb processing is a unique pharmaceutical technique, which can turn Chinese medicinal materials into decoction pieces using processing methods like stir-frying, steaming, and frying with sand or wheat bran [10]. Decocting pieces are the main form prescribed in TCM clinics [1]. The pharmacological actions and energy properties (nature, flavor, and channel tropism) might be changed after processing, side effects and disagreeable odors can be eliminated. It is essential to use the proper processing method to ensure the quality and safety of traditional Chinese medicine decocting pieces. Steaming or stir-frying with salt can change the activity direction of Chinese herbal medicines and can also improve their efficacy [11–14].

The commercially available products of *Morinda officinalis* in herbal markets are *M. officinalis* without wood (MO) and salt-processed products (SMO). It was revealed that MO extract exerts tonifying kidney and supporting yang effects by regulating the functions of the hypothalamus-pituitary-adrenal axis [15, 16]. The medicinal efficacy of MO and SMO is different because of their different processing methods. After being salt processed, the effects of strengthening kidney-yang can be dramatically enhanced. The chemical composition changes during processing, for example, glycosides can be hydrolyzed into aglycone or converted into other constituents, the contents of toxic ingredients can be decreased or converted into other constituents [17, 18].

Monotropein, which possess anti-inflammatory, analgesia, and antiosteoporotic activities, is the main iridoid glycoside isolated from *M. officinalis* [19, 20]. Anthraquinones, especially alizarin-anthraquinones, like rubiadin and rubiadin 1-methyl ether, can inhibit osteoclastic bone resorption in vitro and invigorate the kidney-yang [21–24]. Therefore, in the present study, we chose monotropein, rubiadin, and rubiadin 1-methyl ether as the representative compounds to explain the effects of the different processing methods on the pharmacokinetics of *M. officinalis*.

There have been reports on the pharmacokinetics or tissue distribution of iridoids and oligosaccharides using HPC-DAD and LC-MS [25–28]. However, the abovementioned studies paid more attention to pure iridoid, inulin-type fructo-oligosaccharides and neglected interactions among iridoids and other compounds. There still has been no report about the pharmacokinetics and tissue distribution characteristics of alizarin-anthraquinones in *M. officinalis*. Also, the effect of processing methods on the pharmacokinetics, bioavailability, and tissue distribution characteristics of iridoids and anthraquinones has barely been reported.

In this study, we developed a selective and sensitive UPLC-MS/MS method for simultaneous determination of monotropein, rubiadin, and rubiadin 1-methyl ether in rat plasma and tissues. The analysis method was successfully applied to the pharmacokinetics study of MO and SMO extracts.

## 2. Materials and Methods

### 2.1. Materials and Chemicals

*M. officinalis* were collected from Gaoliang County, Guangdong Province, and identified by Prof. Feng Li of the Liaoning University of Traditional Chinese Medicine. For reference substance, rubiadin (J0111AS), rubiadin 1-methyl ether (J0307AS), monotropein (00605AS) were purchased from Meilun Biological Technology Co. Ltd. (Shanghai, China). The internal standards (ISs) geniposide (1203A023) and 1,8-dihydroxyanthraquinone (100398–200701) were provided by the National Institute for Food and Drug Control (Beijing, China).

Merck KGaA (Darmstadt, Germany) supplied LC-MS-grade formic acid and acetonitrile (HPLC-grade). The ultrapure water was generated with a Milli-Q water purification system (18.2 MΩ, Millipore, Billerica, USA). Other reagents were of analytical grade from Tianjin Kermol Chemical Reagent Co., Ltd. (Tianjin, China) and Waters Xevo TQD Mass Spectrometer (Massachusetts, USA).

### 2.2. Animals

Sprague-Dawley (SD) rats (200 ± 20 g, weight) were purchased from Changsheng Biotechnology Co. Ltd. (License No. SCXK (Liao) 2015-0001, Benxi, Liaoning Province). The experimental animals were kept at 25 ± 2°C and 60% ± 10% humidity with a 12 h light/dark cycle. Water and chow were provided ad libitum. All animal pharmacological experiments followed the ethical regulations of the Liaoning University of Traditional Chinese Medicine.

### 2.3. LC-MS Analytical Conditions

For LC-MS/MS analysis, data acquisition and instrument control were performed using MassLynx 4.1 software (Waters Corp., Milford, MA, USA). The analysis column was an Acquity UPLC HSS T3 column (100 mm × 2.1 mm, 1.8 *μ*m, Waters) with a temperature of 40°C. The mobile phase was acetonitrile containing 0.1% formic acid (A) and 0.1% formic acid aqueous solution (B). The elution gradient was 0.00–0.50 min with 2% B; 0.51–5.00 min with 2%–30% B; and 5.01–7.00 min with 40% B. The flow rate was 0.2 mL/min.

**Figure 1:**
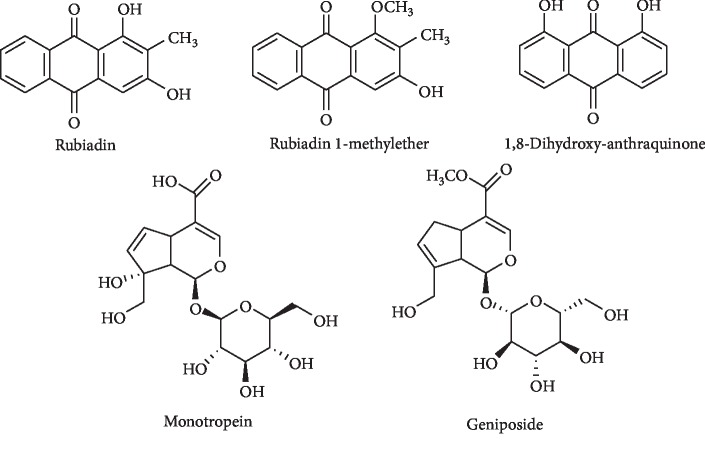
Structure of standard and internal compounds.

The Waters triple quadruple mass spectrometer (Xevo TQD, Waters Corp., Milford, MA, USA) equipped with an electrospray ionization source (ESI) was used in negative ion mode. The desolvation gas was nitrogen with a flow rate of 500 L/h at a temperature of 250°C. All detected compounds were measured in multiple reaction monitoring (MRM) mode.  Figure 1 shows the chemical structures of the three analytes and the internal standards.

The precursor and product ion pairs for MRM were *m/z* 389.01 ⟶ 191.01 for monotropein (collision energy 15 eV); *m/z* 253.05 ⟶ 195.21 for rubiadin (collision energy 40 eV); *m/z* 267.07 ⟶ 252.04 for rubiadin 1-methyl ether (collision energy 18 eV); *m/z* 387.03 ⟶ 225.03 for IS geniposide (collision energy 8 eV); and *m/z* 238.94 ⟶ 166.99 for IS 1,8-dihydroxyanthraquinone (collision energy 35 eV).

### 2.4. Preparation of Reference Substances

Monotropein, rubiadin, and rubiadin 1-methyl ether were dissolved in methanol to prepare a stock solution with a concentration of 0.5 mg/mL. The stock solution was diluted with methanol to get the appropriate concentrations for the working standard solutions. The IS stock solution (1 mg/mL) and IS working solution (200 ng/mL) were also prepared in methanol, as described above. All prepared solutions were stored at 4°C before use.

The preparation of calibration standards was done as follows: The appropriate working solution was spiked into blank plasma or tissues to obtain concentrations of 75∼0.047 *μ*g/mL for monotropein, 396∼1.54 *μ*g/mL for rubiadin, and 596∼1.16 *μ*g/mL for rubiadin 1-methyl ether. The QC concentrations for monotropein were 0.14 *μ*g/mL, 2 *μ*g/mL, and 28.1 *μ*g/mL; for rubiadin, they were 1.6 *μ*g/mL, 50.6 *μ*g/mL, and 712.5 *μ*g/mL; and for rubiadin 1-methyl ether, they were 3.9 *μ*g/mL, 54.6 *μ*g/mL, and 765 *μ*g/mL. The QC samples used for the recovery, matrix effect, intra- and interday accuracy, precision, and stability studies were prepared in the same way as that of the calibration standards. The solutions were stored for one week at −20°C.

### 2.5. Preparation of MO and SMO Extract

We obtained the products of *M. officinalis* without wood and salt-processed *M. officinalis* by following processing methods.

  MO: steam the clean *Morindae officinalis* Radix for 0.5 h, remove the woody cores while hot, cut into sections, and dry.
  SMO: steam the clean *Morindae officinalis* Radix with salt water for 4.0 h, until it is steamed thoroughly, remove the woody cores while hot, cut into sections and dry.

Then, MO and SMO were refluxed with 80% ethanol three times for 1 h each to make a combined filtrate. Then, the extracts were concentrated to 4 g·mL^−1^.

The contents of the three constituents in the MO and SMO extracts were detected by the same UPLC-MS/MS method, including the same column, mobile phase system, and column temperature as used for the pharmacokinetics and tissue distribution study. The contents of monotropein, rubiadin, and rubiadin 1-methyl ether in the MO and SMO extracts were 6.44, 0.088, and 0.174 and 3.95, 0.091, and 0.178 mg/g, respectively. The concentrated extract was redissolved in distilled water.

### 2.6. Preparation of Biological Samples

10 *μ*L of IS working solution and 400 *μ*L of methanol were added to 100 *μ*L plasma. The solution was vortexed for 10 min and then centrifuged at 12,000 rpm for 5 min. The supernatant was shifted to another centrifugation tube and dried with nitrogen at 37°C. The residue was redissolved in 100 *μ*L acetonitrile and centrifuged for 5 min at 12,000 rpm. Five microliters of supernatant was drawn and analyzed by UPLC-MS/MS.

The whole tissue was cut into pieces and homogenized with ice-cold 0.9% (m/v) sodium chloride solution (1 : 9, w/v). Using 50 *μ*L of tissue homogenate, the tissue samples were prepared in the same way as the plasma samples.

### 2.7. Method Validation

#### 2.7.1. Specificity

Six individual sources of blank plasma were used to measure the specificity of this analysis method. Meanwhile, we also analyzed the lower limit of quantification (LLOQ) of samples, the IS in blank plasma samples, and plasma samples within 2 h of being subjected to the MO extract (40 g/kg).

#### 2.7.2. Calibration Curve and Sensitivity

The peak-area ratio of the compounds to the IS was plotted to obtain the linearity. The weighted least-squares linear regression (weighting factor 1/*X*^2^) was applied to determine the regression equations. The lowest concentration in the calibration curve was determined as the LLOQ with signal-to-noise of ≥10.

#### 2.7.3. Precision, Accuracy, and Stability

Through evaluating three levels of QC samples (*n* = 5) after one day and then for three days in a row, we obtained the intra- and interday precision, along with the accuracy data. The permitted range was within 15%.

The QC samples used for the recovery QC samples were used to judge the stability, and the storage conditions were as follows: (1) freeze-thaw stability, (2) at room temperature for 4 h, and (3) put into the autosampler for 8 h, and (4) at −20°C for 30 days. The permitted range was within 15%.

#### 2.7.4. Extraction Recovery and Matrix Effect

Three different QC sample concentrations were used for the recovery experiment. The absolute extraction recovery was measured as the ratio of the QC sample concentration extracted from the blank plasma/tissue to those in the QC samples.

The peak areas of the biosamples extracted in blank plasma/tissue versus those dissolved in methanol solution were measured to obtain the matrix effect.

### 2.8. Analysis of Pharmacokinetics

The SD rats (6, male) were housed at 22 ± 2°C and fasted for 12 h with drinking water available ad libitum. MO and SMO ethanol extract were orally administered at a dose of 40 g/kg (equivalent to 6.44 and 3.95 mg/kg of monotropein, 0.088 and 0.091 mg/kg of rubiadin, and 0.174 and 0.178 mg/kg of rubiadin 1-methyl ether). Blood samples (0.5 mL) were collected from the venous plexus of the eye socket at 0.083, 0.25, 0.5, 1.0, 1.5, 2.0, 2.5, 4.0, 6.0, 12.0, and 24 h under anesthesia. Then, the blood samples were centrifuged at 4500 rpm for 10 min, and then the plasma was immediately transferred to new tubes and stored at −80°C until analysis. The pharmacokinetic parameters were calculated using DAS software (3.0 version, China Food and Drug Administration) based on noncompartmental method. All data were recorded as mean ± SD.

### 2.9. Analysis of Tissue Distribution

MO and SMO ethanol extract (40 g/kg) was orally administered to SD rats (weight, 180–220 g, *n* = 6), at 0.25, 1, 3, and 6 h, respectively. Heart, liver, spleen, stomach, kidney, brain, and small intestine tissues were extracted, washed with saline, blotted with filter paper, weighed, and then stored at −80°C.

## 3. Results and Discussion

### 3.1. Liquid Chromatography Optimization

We tested mobile phase and gradient elution programs to determine the best chromatographic performance. With the mobile phase of water (containing 0.1% formic acid) and acetonitrile (containing 0.1% formic acid), the responses of the three components and two ISs were considerably better. To reach a better and more rapid separation effect, we optimized the gradient elution program.

### 3.2. Mass Spectrometry Optimization

We carried out both positive and negative modes in mass spectrometry. Three components and the ISs all had high responses under the negative mode. The MS/MS transitions and parameters are given in Table 1.

### 3.3. Extraction Procedure Optimization

Protein precipitation (PP) was used for the pretreatment of biological samples. We applied methanol and acetonitrile for PP. The samples had better peak shapes and recovery after being treated by acetonitrile, so we chose acetonitrile for further PP in this study.

### 3.4. Method Validation

#### 3.4.1. Specificity

**Figure 2:**
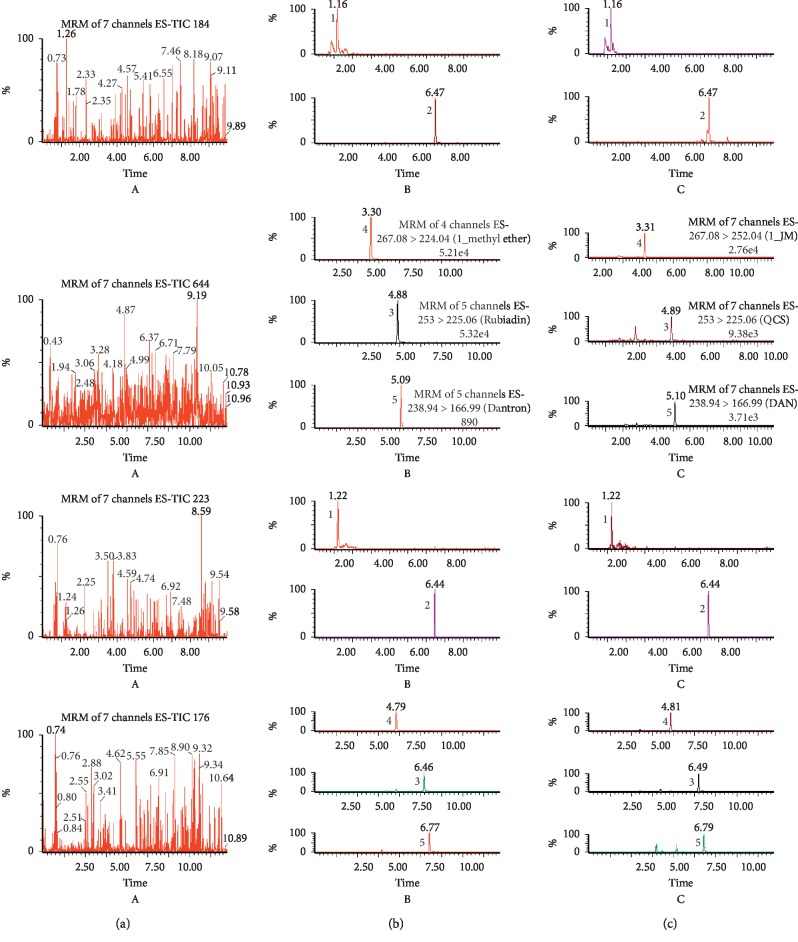
Multiple reaction monitoring (MRM) chromatograms of monotropein, rubiadin, rubiadin 1-methyl ether, and the IS in rat plasma: (a) blank plasma samples, (b) blank plasma samples spiked with three analytes at the lower limit of quantification (LLOQ), and (c) plasma samples at 2 h after the oral administration of *M. officinalis* extract (40 g/kg). (1) Monotropein, (2) geniposide, (3) rubiadin, (4) rubiadin 1-methyl ether, and (5) 1,8-dihydroxyanthraquinone.

The MRM chromatograms of three components and the ISs are shown in Figure 2. The peak separation was better for the three components and the ISs under the established UPLC-MS/MS conditions, with no significant interference and no cross interference.

#### 3.4.2. Linearity and Sensitivity

The internal standard method was applied for the establishment of calibration curves, which showed good linearity (*r*^2^ > 0.9907) in the linear ranges. The regression equations, correlation coefficients, linear ranges, and LLOQs of the three components in the plasma and tissues are listed in Table 2.

#### 3.4.3. Precision, Accuracy, and Stability

The range of intra and interday precisions was 0.21% to 5.96%, and the accuracy range was from −8.06% to 1.33% for QC samples in plasma and tissues. These values are in the acceptable range, and the results are shown in Table 3.

Table 4 shows the results for the stability of the plasma and tissue samples in different conditions. They are also in the acceptable range.

#### 3.4.4. Extraction Recovery and Matrix Effect

The range of extraction recoveries was 80.07∼98.64% for the three QC sample levels (Table 5). These data indicate that the sample treatment method is reasonable. No significant effect of endogenous substances was identified.

### 3.5. Pharmacokinetics Study

**Figure 3:**
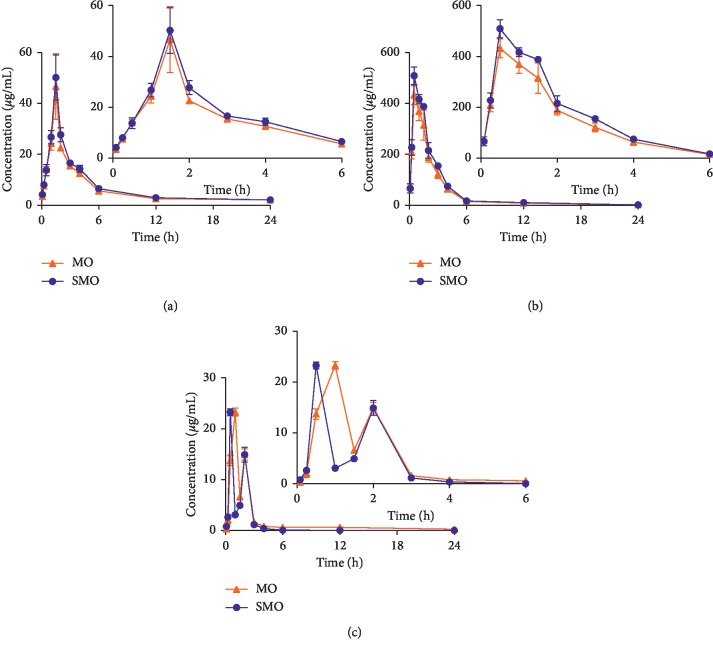
Mean (±SD, *n* = 6) plasma concentrations of rubiadin, rubiadin 1-methyl ether, and monotropein after oral administration of *M. officinalis* without wood (MO) and salt-processed product (SMO) extracts (40 g/kg). (a) Rubiadin, (b) rubiadin 1-methyl ether, and (c) monotropein.

After oral administration of MO and SMO extract (40.0 g/kg), we successfully determined the concentrations of monotropein, rubiadin, and rubiadin 1-methyl ether in rat plasma using the established method.  Figure 3 shows the mean plasma concentration-time profiles.  Table 6 displays the pharmacokinetic parameters.

The concentration of monotropein in SMO reached *C*_max_ in 0.5 h, whereas the *T*_max_ of monotropein in the MO groups was 1.0 h, which indicates that monotropein was quickly adsorbed into the blood after oral administration. The *T*_max_ for rubiadin was 1.5 h, longer than that for rubiadin 1-methyl ether, whereas the *C*_max_ for rubiadin 1-methyl ether was the highest, especially in the SMO groups. In the plasma concentration-time curves of monotropein, an obvious double-peak phenomenon was found, which is related to enterohepatic recirculation. The pharmacokinetics properties shown in the present assay could be helpful for further studies on the pharmacokinetics of MO extract and further applications in different processing methods.

### 3.6. Tissue Distribution

**Figure 4:**
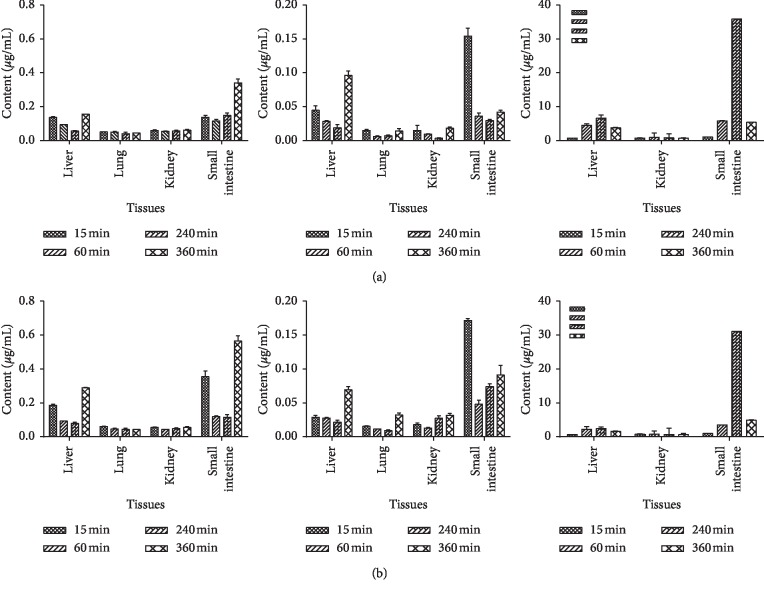
Concentrations of rubiadin-1-methyl ether, rubiadin, and monotropein in rat tissues at 15 min, 60 min, 240 min, and 360 min after intragastric administration of MO and SMO extracts (mean ± SD, *n* = 6). (a) Tissue distribution of the MO extract and (b) tissue distribution of the SMO extract. (1) Rubiadin-1-methyl ether, (2) rubiadin, and (3) monotropein.

The concentrations of monotropein, rubiadin, and rubiadin 1-methyl ether in the liver, kidney, lung, and small intestine were determined at 0.25, 1, 3, and 6 h after administration. The results are shown in Figure 4. All compounds were detected in tissues, except for monotropein, which could not be quantified in the lung tissue. This result indicates that monotropein might be rapidly transformed into its metabolites in the lungs, or the content of monotropein in the lung was lower. Meanwhile, all three components had higher concentrations in the small intestine, especially rubiadin and rubiadin 1-methyl ether in the SMO groups.

## 4. Conclusion

In this study, we established an efficient and accurate UPLC-MS/MS method for the determination of three components from MO and SMO in plasma and tissues after oral administration in rats. Meanwhile, this is the first simultaneous determination of monotropein, rubiadin, and rubiadin-1-methyl ether in rat plasma and tissues. The study examined the pharmacokinetics and tissue distribution. The information found might partially illustrate their metabolic mechanisms in vivo, as well as provide a scientific basis for the strengthening of kidney-yang using the salt-processed principle of *M. officinalis* because of better bioavailability.

## Figures and Tables

**Table 1:** The MS/MS transitions and energy parameters of analytes and IS.

Compound	[M-H]^−^ (*m*/*z*)	Quantitative ion (*m*/*z*)	Cone voltage (V)	Collision energy (eV)
Rubiadin	253.05	225.06	58	40
Rubiadin 1-methyl ether	267.06	252.04	50	18
Monotropein	389.11	191.03	40	15
1,8-Dihydroxyanthraquinone	239.04	211.04	40	35
Geniposide	387.13	225.07	36	8

**Table 2:** Calibration curves and LLOQs of components in rat plasma and tissues.

	Components	Calibration curves	*r* ^2^	Range (*μ*g/mL)	LLOQ (*μ*g/mL)
Plasma	Rubiadin	*Y* = 3.32809*X* + 0.40361	*r* ^2^ = 0.9936	1.2∼950	1.2
	Rubiadin 1-methyl ether	*Y* = 1.08619*X* + 1.03779	*r* ^2^ = 0.9919	1.3∼1020	1.3
	Monotropein	*Y* = 0.0256*X* − 0.0026	*r* ^2^ = 0.9943	0.047∼37.5	0.047

Liver	Rubiadin	*Y* = 4.4365*X* + 0.629	*r* ^2^ = 0.9928	0.07∼6.18	0.07
	Rubiadin 1-methyl ether	*Y* = 5.0622*X* + 0.7667	*r* ^2^ = 0.9911	0.05∼4.66	0.05
	Monotropein	*Y* = 0.0315*X* − 0.0741	*r* ^2^ = 0.9915	0.47∼37.5	0.47

Small intestine	Rubiadin	*Y* = 3.4909*X* − 2.2398	*r* ^2^ = 0.9907	0.07∼12.375	0.07
	Rubiadin 1-methyl ether	*Y* = 6.4061*X* − 0.1149	*r* ^2^ = 0.9926	0.092∼9.2	0.092
	Monotropein	*Y* = 0.0214*X* − 0.0251	*r* ^2^ = 0.9969	0.47∼37.5	0.47

Kidney	Rubiadin	*Y* = 3.5819*X* − 0.9479	*r* ^2^ = 0.9938	0.07∼6.18	0.07
	Rubiadin 1-methyl ether	*Y* *=* *5.3299X* − 0.6431	*r* ^2^ = 0.9968	0.05∼4.66	0.05
	Monotropein	*Y* = 0.0333*X* − 0.0156	*r* ^2^ = 0.9997	0.47∼37.5	0.47

Lung	Rubiadin	*Y* = 4.4389*X* − 0.7058	*r* ^2^ = 0.9966	0.07∼6.18	0.07
	Rubiadin 1-methyl ether	*Y* = 8.7095*X* − 2.3863	*r* ^2^ = 0.9919	0.05∼4.66	0.05

**Table 3:** Precision and accuracy for monotropein, rubiadin, and rubiadin 1-methyl ether in biological samples.

Matrix	Compounds	Conc. (*μ*g/mL)	Intra-day (*n* = 5)		Inter-day (*n* = 5)	
			Precision (RSD%)	Accuracy (RE%)	Precision (RSD%)	Accuracy (RE%)
Plasma	Rubiadin	3.6	1.23	−8.06	0.21	−3.06
		50.6	2.77	−2.92	2.85	−2.75
		712.5	5.96	−2.01	2.79	−4.15
	Rubiadin 1-methyl ether	3.9	4.91	−7.44	3.42	−2.82
		54.6	2.33	−0.33	1.73	−2.44
		765	2.28	−5.45	3.33	−3.53
	Monotropein	0.14	3.51	−1.42	3.27	−2.13
		2	1.25	−3.4	1.93	−0.9
		28.1	2.79	−3.36	0.76	−2.9
Small intestine	Rubiadin	0.21	4.44	−1.43	4.75	−2.86
		0.98	4.43	−0.1	4.62	1.12
		4.63	4.55	−0.41	1.15	−0.82
	Rubiadin 1-methyl ether	0.15	1.66	−0.67	1.12	−2.67
		0.72	5.83	−0.69	5.05	0.14
		3.49	5.55	−0.14	2.85	0.06
	Monotropein	1.41	5.55	−0.07	1.72	−0.71
		6.2	1.63	−1.29	2.48	0.16
		28.13	3.49	−0.64	3.41	−0.25
Liver	Rubiadin	0.21	3.43	−0.48	1.9	−0.48
		0.98	1.3	−0.51	2.89	−0.41
		4.63	2.5	−0.13	3.33	0.04
	Rubiadin 1-methyl ether	0.15	2.42	−4	4.62	1.33
		0.72	1.57	−0.28	5.56	−1.25
		3.49	2.27	−0.11	2.54	−0.2
	Monotropein	1.41	4.36	−0.35	4.75	−0.78
		6.2	1.32	−0.81	2.2	0.48
		28.13	5.95	−2.03	3.82	0.03
Kidney	Rubiadin	0.21	1.86	−0.05	5.96	−3.81
		0.98	1.56	−0.51	3.71	−0.51
		4.63	3.23	−0.11	2.36	−0.13
	Rubiadin 1-methyl ether	0.15	3.33	−2	4.18	−2.67
		0.72	3.06	−0.56	4.35	−0.28
		3.49	2.17	−0.14	4.35	0.09
	Monotropein	1.41	1.5	−0.78	2.54	−0.64
		6.2	3.74	−1.29	3.5	−0.81
		28.13	1.21	0.02	3.63	−0.5
Lung	Rubiadin	0.21	1.2	−0.38	2.15	0.48
		0.98	2.88	−0.51	1.8	0.2
		4.63	5.93	−0.04	3.33	−0.17
	Rubiadin 1-methyl ether	0.15	5.04	−2.67	0.6	−1.33
		0.72	1.33	−0.28	1.59	0.28
		3.49	5.52	−0.06	1.72	0.06

**Table 4:** Stability of the three components in rat plasma and tissues (*n* = 5).

Matrix	Compounds	Conc (*μ*g/mL)	At 25°C for 12 h		−20°C for 30 days		Autosampler at 4°C for 24 h		Freeze–thaw cycles	
				RSD%		RSD		RSD		RSD
			RE%		RE%	%	RE%	%	RE%	%
Plasma	Rubiadin	3.6	−6.23	4.18	−3.16	2.13	−1.72	3.13	−0.92	4.22
		50.6	−1.62	3.86	−2.38	1.45	−0.97	4.13	−1.35	1.45
		712.5	−4.1	4.41	−2.46	3.81	−1.76	3.56	−2.34	6.27
	Rubiadin 1-methyl ether	3.9	−1.79	1.63	−3.26	2.36	−1.49	2.48	−0.94	3.85
		54.6	−4.21	3.41	−0.23	3.42	−1.65	3.53	−1.35	3.18
		765	−5.1	2.81	−0.86	3.13	−3.14	1.73	−3.71	2.45
	Monotropein	0.14	−0.14	3.21	−1.21	2.51	−2.39	2.46	−1.95	2.99
		2	−0.13	2.41	−0.24	2.91	−0.46	3.74	−0.52	4.82
		28.1	−0.21	3.92	−1.36	4.82	−1.23	3.69	−2.89	3.91

Small intestine	Rubiadin	0.21	−0.07	2.59	−0.35	1.98	−0.64	5.7	−0.45	3.54
		0.98	−0.57	2.96	−2.71	1.14	−1.65	5.79	−0.38	3.85
		4.63	−1.47	5.43	−0.87	8.51	−1.67	2.97	0.39	2.68
	Rubiadin 1-methyl ether	0.15	−0.69	7.53	−1.25	0.65	−0.42	2.74	−6.57	8.41
		0.72	−0.85	6.41	−0.25	4.23	−0.62	2.19	−0.57	1.88
		3.49	0.25	2.47	1.35	2.49	0.48	7.29	−0.74	8.82
	Monotropein	1.41	−0.63	3.61	−0.85	1.58	−0.35	4.86	−0.99	1.25
		6.2	−1.41	5.93	−0.71	3.87	−0.66	5.87	0.15	6.02
		28.13	−2.27	4.86	−0.12	4.64	−0.01	6.84	−0.01	2.57

Liver	Rubiadin	0.21	−0.52	1.38	−0.54	1.28	0.26	1.07	−5.57	6.37
		0.98	−2.43	0.86	−0.8	8.16	−0.33	2.08	0.25	4.23
		4.63	−0.57	2.36	1.57	5.37	−2.36	2.97	0.27	3.67
	Rubiadin 1-methyl ether	0.15	−2.1	5.64	−0.58	3.76	0.54	2.97	−1.52	5.24
		0.72	−3.42	6.84	−0.45	1.53	−3.81	7.23	−5.42	4.27
		3.49	0.42	2.23	−2.58	2.45	−0.66	3.88	−0.68	2.68
	Monotropein	1.41	−0.85	0.41	−1.84	6.21	−2.98	1.78	−1.77	3.72
		6.2	0.56	1.68	−0.52	4.2	0.23	2.87	−0.84	2.98
		28.13	−1.71	3.69	−0.65	3.51	−3.16	2.97	0.09	1.96

Kidney	Rubiadin	0.21	−0.42	0.32	−0.54	6.45	−0.45	7.95	−0.57	1.87
		0.98	−0.38	0.98	−2.45	2.76	−6.72	4.56	−1.57	6.41
		4.63	−0.17	3.76	−0.67	0.64	1.46	2.17	0.38	5.13
	Rubiadin 1-methyl ether	0.15	0.35	2.94	−5.1	2.76	−0.36	1.56	0.15	2.51
		0.72	0.24	1.9	−2.49	3.57	−0.68	2.35	−0.54	5.42
		3.49	−3.58	4.35	0.54	1.94	−0.36	3.56	−1.57	4.23
	Monotropein	1.41	−0.28	6.72	−0.86	1.62	−0.18	3.5	0.24	3.69
		6.2	−0.67	6.42	−0.46	7.56	−0.57	5.44	0.07	4.29
		28.13	−0.68	0.34	−0.27	6.52	0.25	2.34	−0.57	2.86

Lung	Rubiadin	0.21	0.65	0.86	0.42	3.87	−0.24	5.44	−0.38	5.71
		0.98	0.38	3.57	0.48	0.68	−0.54	3.46	−1.57	5.74
		4.63	0.68	6.84	−5.71	0.42	−0.57	3.55	0.35	6.7
	Rubiadin 1-methyl ether	0.15	−0.57	6.42	−1.54	0.35	−8.51	7.62	−2.57	3.85
		0.72	−5.71	2.43	−0.55	7.36	−0.65	3.57	−4.85	4.42
		3.49	−0.06	2.46	0.05	4.51	−2.42	6.58	−0.53	2.46

**Table 5:** Extraction recoveries in rat plasma and tissues (*n* = 5).

Matrix	Compounds	Conc. (*μ*g/mL)	Recoveries (%)	RSD (%)
Plasma	Rubiadin	3.6	88.34	7.03
		50.6	95.08	1.89
		712.5	96.39	0.93
	Rubiadin 1-methyl ether	3.9	81.89	3.24
		54.6	93.42	3.13
		765	94.05	1.44
	Monotropein	0.14	95.51	3.66
		2	91.97	3.88
		28.1	97.60	1.10

Small intestine	Rubiadin	0.21	85.24	3.91
		0.98	91.09	4.05
		4.63	96.21	1.15
	Rubiadin 1-methyl ether	0.15	92.44	2.20
		0.72	91.39	8.03
		3.49	86.97	3.60
	Monotropein	1.41	98.27	1.83
		6.2	98.64	1.03
		28.13	92.95	0.02

Liver	Rubiadin	0.21	89.37	2.15
		0.98	88.98	1.13
		4.63	94.16	2.78
	Rubiadin 1-methyl ether	0.15	88.89	7.21
		0.72	85.97	3.28
		3.49	90.84	1.87
	Monotropein	1.41	97.07	3.25
		6.2	97.67	1.61
		28.13	96.41	0.91

Kidney	Rubiadin	0.21	88.73	4.56
		0.98	82.07	8.44
		4.63	93.59	4.07
	Rubiadin 1-methyl ether	0.15	88.89	4.26
		0.72	93.61	6.61
		3.49	89.98	3.55
	Monotropein	1.41	93.95	4.96
		6.2	93.41	4.95
		28.13	94.47	0.87

Lung	Rubiadin	0.21	80.63	2.46
		0.98	80.07	6.59
		4.63	83.97	1.47
	Rubiadin 1-methyl ether	0.15	91.11	3.30
		0.72	86.67	5.77
		3.49	88.96	5.08

**Table 6:** Pharmacokinetic parameters of the three components in rat plasma (*n* = 6).

Parameters	Units	Mean (rubiadin)		Mean (rubiadin 1-methyl ether)		Mean (monotropein)	
		MO	SMO	MO	SMO	MO	SMO
AUC (0-*t*)	*μ*g/L *∗* h	152.252 ± 7.098	169.355 ± 8.836	1059.49 ± 98.081	1244.329 ± 82.805	44.22 ± 0.932	26.941 ± 1.423
AUC (0-∞)	*μ*g/L *∗* h	153.816 ± 7.169	176.441 ± 18.283	1074.351 ± 102.019	1253.526 ± 84.691	48.952 ± 1.559	27.045 ± 1.338
MRT (0-*t*)	H	6.062 ± 0.272	5.89 ± 0.265	2.908 ± 0.151	2.855 ± 0.15	3.863 ± 0.184	1.686 ± 0.025
*t*1/2*z*	H	3.838 ± 0.196	5.006 ± 3.383	5.379 ± 1.514	4.27 ± 0.865	12.291 ± 2.09	5.611 ± 3.958
*T*_max_	H	1.5 ± 0.00	1.5 ± 0.00	0.5 ± 0.00	0.5 ± 0.00	1.0 ± 0.00	0.5 ± 0.00
*C*_max_	*μ*g/L	46.667 ± 12.941	50.2 ± 8.903	432.367 ± 37.012	509 ± 35.133	23.298 ± 0.764	23.253 ± 0.676
*V/F*	L/g	1.442 ± 0.090	1.579 ± 0.842	0.287 ± 0.067	0.196 ± 0.363	14.447 ± 2.143	12.239 ± 9.027
*CL/F*	L/g·h	0.261 ± 0.012	0.228 ± 0.022	0.037 ± 0.372	0.032 ± 0.210	0.818 ± 0.265	1.482 ± 0.750

## Data Availability

The data used to support the findings of this study are included within the article.
